# Supplementary material for: RAB3 phosphorylation by pathogenic LRRK2 impairs trafficking of synaptic vesicle precursors
Source: bioRxiv. 2023 Jul 25:2023.07.25.550521. Preprint. [Version 1] doi: 10.1101/2023.07.25.550521 (PMC10402060; doi:10.1101/2023.07.25.550521)
Supplement: Supplement 1 [file NIHPP2023.07.25.550521v1-supplement-1.pdf]

bioRxiv preprint doi: https://doi.org/10.1101/2023.07.25.550521; this version posted July 25, 2023. The copyright holder for this preprint (which was not certified by peer review) is the author/funder, who has granted bioRxiv a license to display the preprint in perpetuity. It is made available under aCC-BY 4.0 International license.

Figure S1

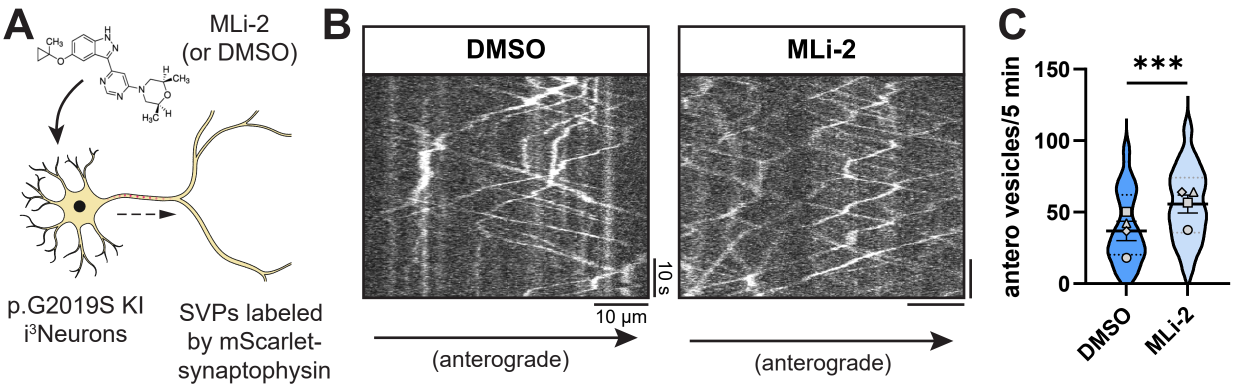

bioRxiv preprint doi: https://doi.org/10.1101/2023.07.25.550521; this version posted July 25, 2023. The copyright holder for this preprint (which was not certified by peer review) is the author/funder, who has granted bioRxiv a license to display the preprint in perpetuity. It is made available under aCC-BY 4.0 International license.

Figure S1. Related to Figure 1. (A) Cartoon depicting p.G2019S KI i^3Neuron treated overnight with DMSO or 100 nM MLi-2. (B) Kymographs of axonal mScarlet-SYP+ vesicles in p.G2019S KI i^3Neurons treated with DMSO or MLi-2. (C) Anterograde flux of SYP+ vesicles in p.G2019S KI i^3Neurons treated with DMSO or MLi-2 (n = 39-41 neurons from 4 independent experiments; ***$p<0.001$; linear mixed effects model). Scatter plot points indicate the means of four independent experiments, and error bars show mean ± SD of these points.

bioRxiv preprint doi: https://doi.org/10.1101/2023.07.25.550521; this version posted July 25, 2023. The copyright holder for this preprint (which was not certified by peer review) is the author/funder, who has granted bioRxiv a license to display the preprint in perpetuity. It is made available under aCC-BY 4.0 International license.

Figure S2

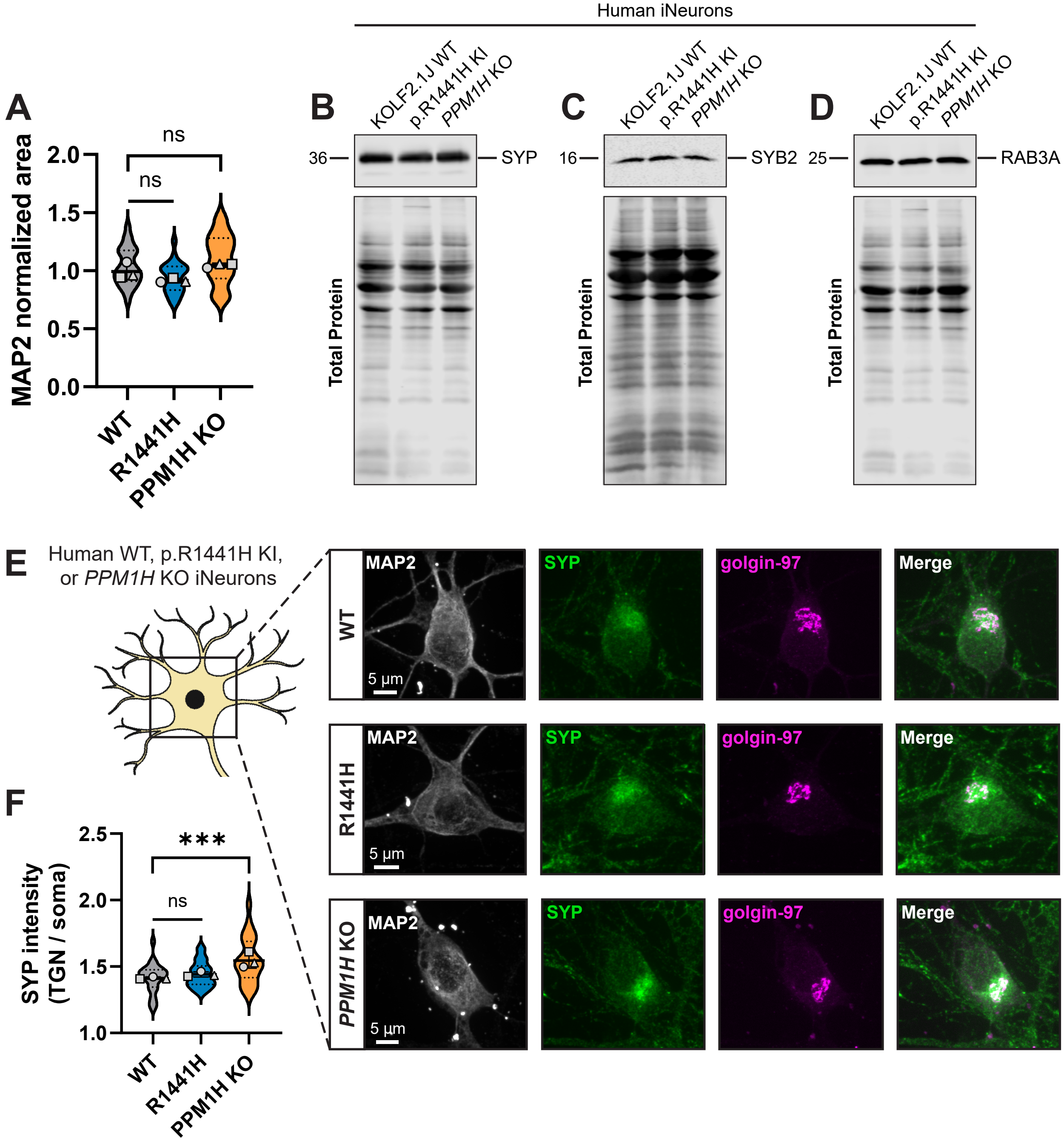

bioRxiv preprint doi: https://doi.org/10.1101/2023.07.25.550521; this version posted July 25, 2023. The copyright holder for this preprint (which was not certified by peer review) is the author/funder, who has granted bioRxiv a license to display the preprint in perpetuity. It is made available under aCC-BY 4.0 International license.

Figure S2. Related to Figure 3. (A) Normalized somal MAP2 area of WT, p.R1441H KI, and *PPM1H* KO iNeurons for dataset shown in Figure 3A-C (n = 24 neurons from 3 independent experiments; ns >0.0540; linear mixed effects model). (B-D) Example total protein stain and immunoblot of SYP (B), SYB2 (C), and RAB3A (D) in DIV21 WT, p.R1441H KI, and *PPM1H* KO iNeurons. (E) Representative images of DIV14 WT, p.R1441H KI, and *PPM1H* KO iNeuron somas, stained for endogenous MAP2, SYP, and golgin-97. (F) Ratio of SYP intensity (mean grey value) co-localized with golgin-97 signal / SYP intensity (mean grey value) of whole soma, in WT, p.R1441H KI, and *PPM1H* KO iNeurons (n = 24 neurons from 3 independent experiments; ns = 0.4638; ***p<0.001; linear mixed effects model). Scatter plot points indicate the means of 3 independent experiments, and error bars show mean ± SD of these points.

bioRxiv preprint doi: https://doi.org/10.1101/2023.07.25.550521; this version posted July 25, 2023. The copyright holder for this preprint (which was not certified by peer review) is the author/funder, who has granted bioRxiv a license to display the preprint in perpetuity. It is made available under aCC-BY 4.0 International license.

Figure S3

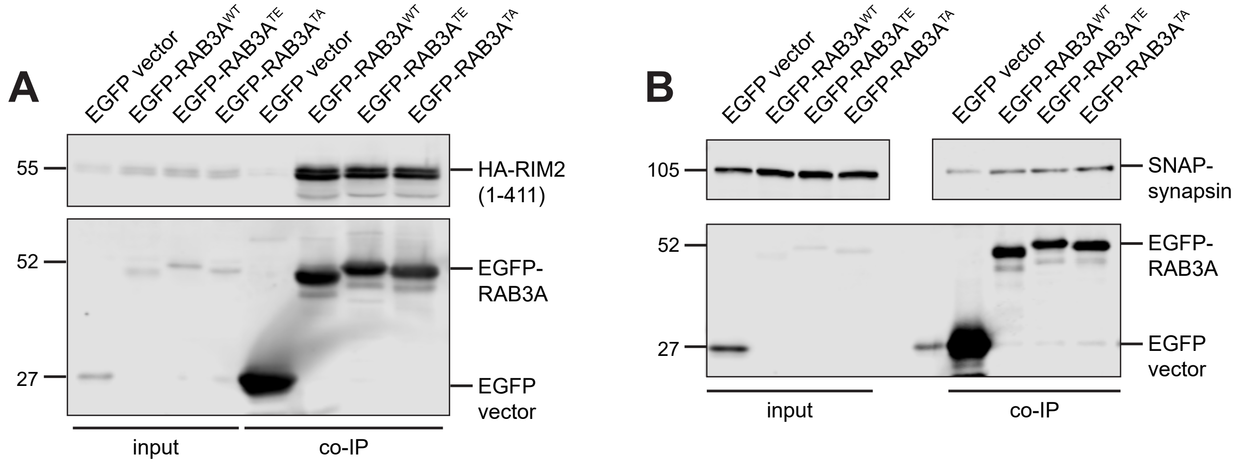

bioRxiv preprint doi: https://doi.org/10.1101/2023.07.25.550521; this version posted July 25, 2023. The copyright holder for this preprint (which was not certified by peer review) is the author/funder, who has granted bioRxiv a license to display the preprint in perpetuity. It is made available under aCC-BY 4.0 International license.

Figure S3. Related to Figure 5. (A) Example immunoblot of RIM2 (first 411 residues) co-immunoprecipitation by $RAB3A^{WT}$, $RAB3A^{TE}$, or $RAB3A^{TA}$, co-expressed in HEK293T cells. (B) Example immunoblot of synapsin co-immunoprecipitation by $RAB3A^{WT}$, $RAB3A^{TE}$, or $RAB3A^{TA}$, co-expressed in HEK293T cells. Upper panel is separated for alignment purposes; no lanes that included sample were excluded. For all co-IP experiments shown, samples were processed and immunoblotted in parallel.

bioRxiv preprint doi: https://doi.org/10.1101/2023.07.25.550521; this version posted July 25, 2023. The copyright holder for this preprint (which was not certified by peer review) is the author/funder, who has granted bioRxiv a license to display the preprint in perpetuity. It is made available under aCC-BY 4.0 International license.

Figure S4

RAB3A binding partners

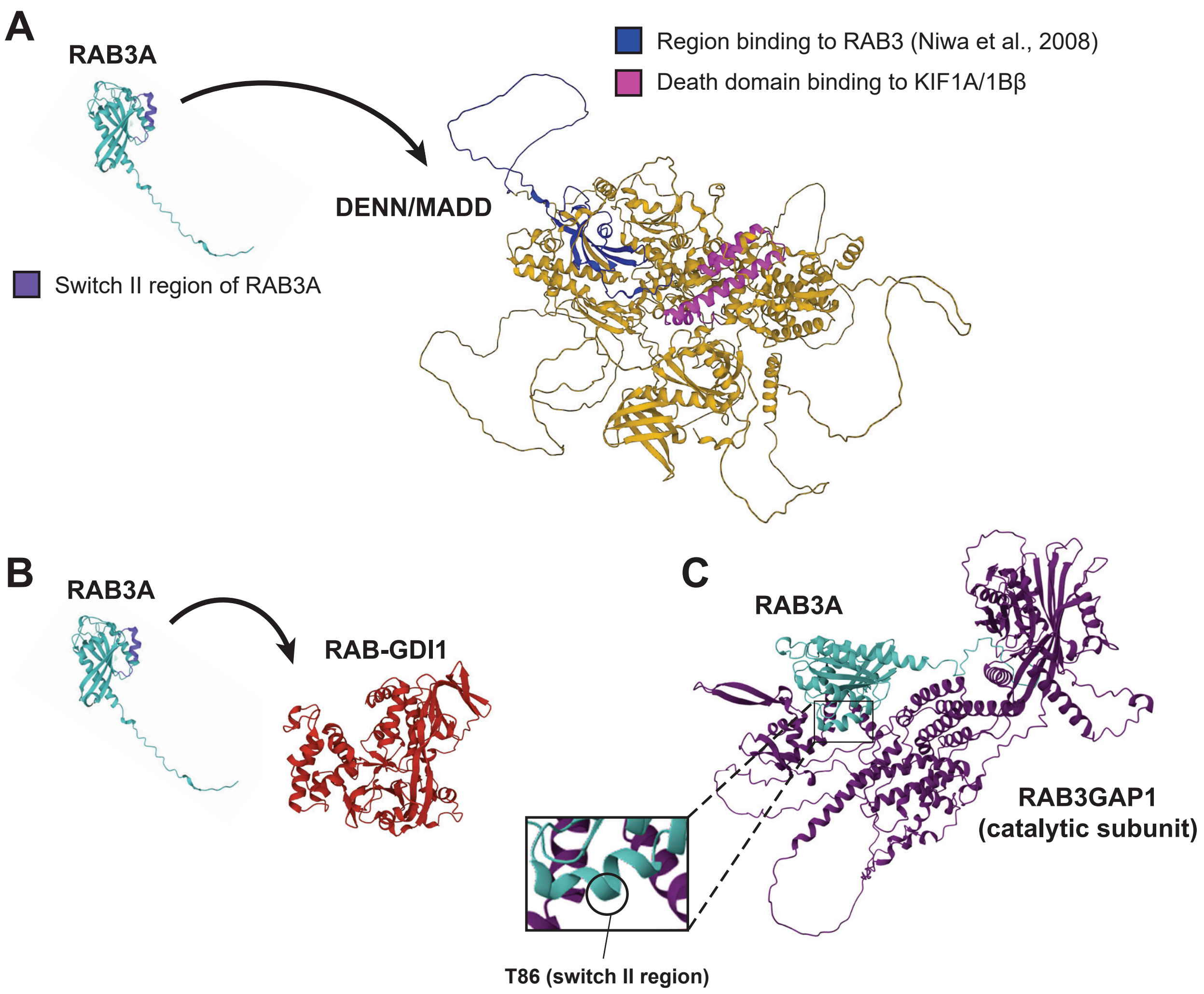

bioRxiv preprint doi: https://doi.org/10.1101/2023.07.25.550521; this version posted July 25, 2023. The copyright holder for this preprint (which was not certified by peer review) is the author/funder, who has granted bioRxiv a license to display the preprint in perpetuity. It is made available under aCC-BY 4.0 International license.

Figure S4. Related to Figures 4 and 5. (A) AlphaFold predictions[40,41] of RAB3A and MADD. Left, annotated in purple: putative switch II region of RAB3A[77]. Right, annotated in blue: the N-terminal 161 residues that were previously shown[16] to be necessary and sufficient for binding to RAB3. Right, annotated in magenta: the death domain toward the C-terminus of MADD that has been shown to be the motor-binding region[16,18] (B) AlphaFold predictions[40,41] of RAB3A and RAB-GDI1. (C) AlphaFold-Multimer[40,41,55–57] prediction of complex formed by RAB3A and RAB3GAP1, the catalytic subunit of RAB3GAP. ipTM + pTM score for this prediction: 0.79404.
